# The Near‐Absence of Isolated Cleft Palate in a Surgical Mission Cohort in Ethiopia: Implications for Infant Mortality and Disease Recognition

**DOI:** 10.1002/cga.70072

**Published:** 2026-08-12

**Authors:** Toshiro Kibe, Kazuhide Nishihara, Kiyohide Ishihata, Aiko Kurosaka, Yuhei Kamikuri, Teruyuki Niimi, Nagato Natsume, Hideto Saijo

**Affiliations:** ^1^ Department of Oral and Maxillofacial Surgery, Field of Oral Maxillofacial Rehabilitation, Developmental Therapeutics Course, Graduate School of Medical and Dental Sciences Kagoshima University Kagoshima Japan; ^2^ Department of Oral Surgery Okinawa Red Cross Hospital Okinawa Japan; ^3^ Division of Research and Treatment for Oral and Maxillofacial Congenital Anomalies, School of Dentistry Aichi Gakuin University Nagoya Aichi Japan

**Keywords:** cleft lip, cleft palate, Ethiopia, global surgery, medical missions, survivorship bias

## Abstract

In Ethiopia, cleft lip and/or palate is a major public health concern. In this study, we compiled clinical statistics on patients who presented during 7 years of surgical medical aid missions and examined possible background factors underlying the striking imbalance in cleft types (i.e., the apparent absence of isolated cleft palate) compared with those in developed countries. All 150 patients examined during surgical medical aid missions conducted between 2013 and 2019 were included. Age, sex, cleft type, surgery performed, and reasons for cancellation were analyzed retrospectively. The mean age of the 150 patients was 4.9 years. Most cases were cleft lip (unilateral cleft lip with or without cleft alveolus) and cleft lip and palate, whereas only two cases (1.3%) were isolated cleft palate. Surgery was performed in 93 cases (62.0%); however, all procedures were cheiloplasty, and palatoplasty was not performed for safety‐related reasons. The surgical cancellation rate was high (38.0%), mainly due to infection and malnutrition. The extreme paucity of patients with isolated cleft palate suggests an “unobserved mortality” in the region. Early death (natural selection) due to feeding difficulties and lack of disease recognition may be the contributing factors. This “cleft‐type imbalance” indicates an urgent need not only to provide surgery, but also to implement early screening and nutritional interventions at the community level.

## 1 Introduction

Cleft lip and/or palate (CL/P) is one of the most common congenital anomalies worldwide [1, 2]. In developing countries, including Ethiopia, due to shortage of specialized surgeons and medical infrastructure, many patients are unable to undergo primary surgery at an appropriate time and live with problems such as social stigma, speech disorders, and feeding difficulties [3, 4, 5]. Our team has continuously conducted medical aid activities to provide plastic reconstructive surgery in Ethiopia since 2013 [6]. Through these activities, a clinical imbalance was observed: while many patients with CL present for care, it is extremely rare to encounter patients with cleft palate only (CP).

This study aimed to compile data on all patients examined over a 7‐year period from 2013 to 2019 to conduct clinical statistical analyses of the distribution of cleft types, patient background, and factors affecting surgical indication, and to consider the factors underlying the observed disparity in cleft patterns.

## 2 Materials and Methods

### 2.1 Study Design and Participants

This was a retrospective study of 150 patients who presented to surgical medical aid activities (surgical mission) in Ethiopia between 2013 and 2019. Patients were recruited through community outreach campaigns conducted by the local GrarBet Hospital across the Butajira region. Since many families in this rural area lack disease awareness and often do not recognize their children's conditions as treatable medical diseases, these active recruitment efforts by the hospital were essential to identify and reach patients who would otherwise remain untreated.

### 2.2 Ethics

This study was conducted with the approval of the Ethics Committee of Kagoshima University (approval number 190019) and clinical research permission from GrarBet Hospital in Ethiopia (approval number 20190228‐01), in accordance with the principles of the Declaration of Helsinki.

### 2.3 Surgical Setting and Team

The mission was conducted at GrarBet Hospital, operated by GrarBet Tehadiso Mahaber (GTM), which covers a population of approximately 1.8 million. The team included oral surgeons, dental anesthesiologists, and a local coordinator.

### 2.4 Protocol for Patient Screening and Safety Management

Patients were recruited by a local coordinator. Preoperative screening was jointly performed by a local pediatrician. Strict “eligibility criteria” were applied including body weight ≥ 6.0 kg, no acute systemic infection (fever or common cold), and no severe developmental delay or complex syndromic conditions. Since chronological age was unreliable due to the absence of official records, such as family registers, and was based on patient/family self‐reports, estimated dental age based on tooth eruption was used to calculate drug dosages and endotracheal tube size. A dental anesthesiologist managed the anesthesia using portable monitors and an oxygen concentrator adapted to frequent power fluctuations.

### 2.5 Data Collection

From local medical records, information on age, sex, diagnosis (cleft‐type classification), procedures performed, and reasons for surgical cancellation was extracted.

### 2.6 Definition: Cleft Types Were Classified as Follows

- Unilateral cleft lip with or without cleft alveolus (UCL ± A)
- Bilateral cleft lip with or without cleft alveolus
- Unilateral cleft lip and palate
- Bilateral cleft lip and palate
- Cleft palate only (CP)
- Submucous cleft palate (SMCP)
- Other (facial cleft and non‐cleft lip/palate disorders)

### 2.7 Statistical Analysis

Data were summarized using descriptive statistics. Continuous variables are presented as mean values and categorical variables as frequencies and percentages. A binomial test was conducted to statistically compare the observed proportion of isolated CP in our cohort with the expected epidemiological prevalence of 20%. Statistical significance was set at *p* < 0.05.

## 3 Results

### 3.1 Patient Background and Diagnosis

During the study period, 150 patients were examined, including 96 males (64%) and 54 females (36.0%) with a male‐to‐female ratio of approximately 1.8:1 (Table 1). The mean age at presentation was 4.9 years, ranging from infants to elderly patients aged 70 years. UCL ± A was the most common type, accounting for 89 (59.3%) cases. Whereas, CP was observed in only two cases (1.3%). This proportion was significantly lower than the expected general prevalence of 20% (*p* < 0.001, binomial test). SMCP was observed in only one case (0.7%).

**TABLE 1:** Patient demographics and diagnosis distribution (*N* = 150).

Diagnosis (cleft type)	Male	Female	Total cases	% of Total	Mean age (years)
UCLP	9	8	17	11.30%	3.1
BCLP	14	2	16	10.70%	1.9
UCL ± A	56	33	89	59.30%	4.5
BCL ± A	11	7	18	12.00%	4.7
CP	2	0	2	1.30%	0.6
SMCP	0	1	1	0.70%	10
Others	4	3	7	4.70%	28.4
Total	96	54	150	100.00%	4.9

Abbreviations: BCL ± A: bilateral cleft lip with/without alveolus; BCLP: bilateral cleft lip and palate; CP: isolated cleft palate; UCL ± A: unilateral cleft lip with/without alveolus; UCLP: unilateral cleft lip and palate; SMCP: submucous cleft palate.

### 3.2 Surgical Implementation Status

Surgery was performed in 93 (62.0%) of the 150 patients examined. Cheiloplasty or lip revision was performed in all the cases. No palatoplasty was performed during this period (Table 2).

**TABLE 2:** Summary of surgical procedures (*N* = 93).

Diagnosis	Cheiloplasty	Lip revision	Total operated
UCLP	14	0	14
BCLP	4	0	4
UCL ± A	63	1	64
BCL ± A	10	1	11
Total	91	2	93

Abbreviations: BCL ± A: bilateral cleft lip with/without alveolus; BCLP: bilateral cleft lip and palate; UCL ± A: unilateral cleft lip with/without alveolus; UCLP: unilateral cleft lip and palate.

### 3.3 Reasons for Surgical Cancellation

A total of 57 patients (38.0%) were deemed ineligible, resulting in surgical cancellation (Table 3). The most common reason was poor general condition, such as upper respiratory infection (URI), fever, or wheezing (29.8%). Cancellation due to malnutrition or low body weight accounted for 21.1% of cases. Other cases were canceled due to comorbidities, such as anemia or heart disease (e.g., ventricular septal defect), or due to diagnoses outside the surgical indication.

**TABLE 3:** Reasons for cancellation (*N* = 57).

Reason categories	Count (*n*)	% of Canceled
URI/fever/poor general condition	17	29.80%
Malnutrition/low body weight	12	21.10%
Not indicated/surgeon's decision	13	22.80%
Other medical (anemia; cardiac; etc.)	15	26.30%
Total	57	100.00%

Abbreviation: URI: upper respiratory infection.

## 4 Discussion

### 4.1 Imbalance in Cleft‐Type Distribution and Epidemiological Discrepancy

The most noteworthy finding in this study was that 1.3% (2/150) of patients had CP only. In the general epidemiology of CL/P, CP accounts for approximately 20%–25% of all cleft cases, and the prevalence of CP in this cohort was markedly lower than the expected incidence. Similarly, reports from other regions of Africa have indicated a low rate of CP surgeries [7]. This disparity in cleft patterns could be attributed to the following two factors.

The first factor is early‐life attrition, leading to “unobserved mortality.” In rural areas with limited medical resources, infants with severe CP experience sucking difficulties and swallowing disorders immediately after birth [8]. If appropriate interventions such as tube feeding are not provided, these children are highly susceptible to death due to severe malnutrition or aspiration pneumonia before reaching the appropriate age for surgery [9, 10]. In other words, the examined cases were likely limited to a subset that survived differential survival pressures, meaning non‐survivors were excluded from our observable population [8].

The second factor is the lack of disease recognition, which intrinsically introduces recruitment bias. In rural Ethiopia, health literacy regarding orofacial clefts remains low. While the facial deformity of CL is highly evident to families and communities, CP is confined to the oral cavity [11]. Consequently, families may not recognize CP as a treatable condition, leading to a systematic recruitment bias where outreach campaigns disproportionately attract patients with visible clefts [12, 13, 14]. Furthermore, while intrinsic genetic or ethnic factors specific to sub‐Saharan African populations might contribute to a lower baseline prevalence of CP, this alone cannot fully explain the extreme disparity observed.

It is also noteworthy that the only two patients with isolated CP in our cohort were male. In general epidemiology, isolated CP shows a female predominance (approximately 2:1). The fact that only male CP patients presented may reflect an additional layer of sampling bias driven by sociocultural factors. In rural Ethiopia, strong patriarchal traditions often prioritize male children for healthcare access and nutritional resources, potentially leaving female infants with CP at an even higher risk of unobserved mortality.

### 4.2 Malnutrition and Barriers to Surgical Intervention

The high cancellation rate (38.0%) supports this hypothesis. Approximately half of the reasons for cancellation were malnutrition and subsequent infections (e.g., upper respiratory infection). This suggests that even patients who survived and presented for care remained in a nutritional crisis. This phenomenon has also been observed in other African regions [15]. Preexisting chronic malnutrition poses a substantial risk to safe surgical intervention [16, 17].

### 4.3 Safety Management and Treatment Strategy

Unlike the medical support activities in Laos and Vietnam, palatoplasties were not performed during this period. The reason was safety management aimed at avoiding the risks of postoperative complications [18]. Palatoplasty in malnourished children carries high risks of postoperative fever, bleeding, wound dehiscence, and postoperative airway obstruction and requires advanced postoperative care [19]. In short‐term medical aid under limited medical resources, intensive postoperative follow‐up could not be adequately implemented locally. Therefore, it was necessary to prioritize safety, avoid life‐threatening complications, and perform triage by selecting cheiloplasty, which can be expected to reliably improve quality of life while avoiding high‐risk procedures.

The strategic use of dental age estimation by dentists in low‐resource settings was crucial [20]. In rural Ethiopia, where birth records are lacking, this method prevented overdose or underdose of anesthetic agents. Furthermore, the high exclusion rate (38%) indicates that a “conservative” approach is necessary to maintain zero mortality in our project in a setting where advanced life‐support measures are unavailable.

### 4.4 Strengths, Limitations, and Future Directions

A primary strength of this study is its provision of essential baseline epidemiological data on CL/P from a rural African cohort over a seven‐year period. However, there are several limitations. First, this was a mission‐based observational study rather than a population‐based demographic survey, limiting the generalizability of the exact prevalence rates. Second, since palatoplasty was not performed during our missions due to safety constraints, there is a theoretical possibility of care‐seeking suppression based on perceived treatment availability. However, we did not pre‐announce the exclusion of palatoplasty to the local communities during recruitment; therefore, it is highly unlikely that patients with CP abstained from seeking care for this reason.

These activities have currently been suspended due to the COVID‐19 pandemic, likely increasing the backlog of untreated patients. Resumption of activities requires not only the provision of surgery but also comprehensive environment building. The epidemiological discrepancies observed in this cohort strongly indicate that surgical missions alone are insufficient. There is an urgent need to establish a systematic neonatal screening system for congenital anomalies, combined with early nutritional interventions and community‐level disease awareness programs.

## Funding

The authors have nothing to report.

## Consent

Verbal informed consent was obtained from all patients or their legal guardians prior to the examinations and surgical procedures. The consent process was facilitated and confirmed by the local physicians in the native language.

## Conflicts of Interest

The authors declare no conflicts of interest.

## Data Availability

The data that support the findings of this study are available on request from the corresponding author. The data are not publicly available due to privacy or ethical restrictions.
